# Sliding Mode Observer with Gain Tuning Method for Passive Interferometric Fiber-Optic Gyroscope

**DOI:** 10.3390/s25113385

**Published:** 2025-05-28

**Authors:** Gabriel F. S. Nunes, João M. S. Sakamoto

**Affiliations:** 1 Graduate Program in Science and Space Technologies, Aeronautics Institute of Technology (ITA), São José dos Campos 12228-900, SP, Brazil; gabrielfsn@ita.br; 2 Division of Photonics, Institute for Advanced Studies (IEAv), São José dos Campos 12228-001, SP, Brazil

**Keywords:** interferometric fiber-optic gyroscope, 3 × 3 optical fiber coupler, sliding mode observer, sliding mode control, control gain

## Abstract

The present work reports the evaluation of a sliding mode observer (SMO) for a passive interferometric fiber-optic gyroscope (IFOG). To achieve that, the experimental setup was designed and evaluated to provide two out-of-phase signals as required by the SMO system employed. This led to the operation of the IFOG-SMO implemented in a passive mode with a 3 × 3 optical fiber directional coupler, which dismissed the use of optical phase modulators, such as piezoelectric or electro-optic devices. Simulations were performed and provided a qualitative analysis of the dependence of the system gain as a function of the sigmoid factor and sample rate, which resulted in a method for tuning the gain value according to the expected input signal. The experimental results show proper working of the IFOG-SMO and the capability for measuring large and small amplitude angular velocities and the expansion of the full scale. This is, to the best of our knowledge, the first time a sliding mode control technique has been employed and evaluated for an interferometric fiber-optic gyroscope.

## 1. Introduction

The sliding mode control is a variable structure nonlinear control technique that provides high robustness to external disturbances, operates under parameters of uncertainty, and has a fast response for nonlinear dynamic plants [1,2]. For this reason, recently, it has been implemented as a control (or observer) strategy in different fields, such as nanopositioning, motor control, and spacecraft attitude stabilization [2,3,4].

In the field of optical interferometry, sliding mode control was first employed in the two-beam Michelson interferometer to achieve highly stabilized operation for displacement measurement, increase the interferometer’s robustness, and avoid signal fading. After a stability analysis, the interferometer was set to operate locked at π/2 and in a small signal regime, such that the phase signal could be directly recovered on the interferometer output [5]. This technique was proven experimentally for the bulk optics Michelson interferometer [5] and fiber-optic Mach–Zehnder interferometer [6].

The sliding mode for interferometry was then expanded for measuring displacement in a large signal regime by employing two outputs (in-phase and quadrature-phase) and nulling the entire interferometer signal [7]. In this case, the relationship between the displacement and the interferometer output became a linear curve, and the signal of interest could be recovered from the control signal. Following those former works, a theoretical analysis with computational simulations was established for the sliding mode control applied to interferometry [8].

The next advance in this field was achieved by developing a sliding mode observer (SMO) to implement a passive Michelson interferometer (passive in this text means the absence of an active control feedback and actuator), which dismissed the phase modulator by taking two out-of-phase outputs and employing the closed-loop inside a digital platform [9].

To continue expanding this field of nonlinear control theory and interferometry, the next possible step is to experiment with a different type of interferometer, the Sagnac interferometer. Despite the similarities between the Sagnac interferometer and the other two, Michelson and Mach–Zehnder, the Sagnac works in a fundamentally different way due to the sharing of the same optical path between both interferometric arms. Because of this, the offset phase shift seen in other interferometers is always zero in an ideal reciprocal assembly of the Sagnac interferometer (classical IFOG minimum configuration) [10]. In reality, there is a nonreciprocal phase drift in the IFOG due to polarization cross-talking, spurious interferometers from back-reflection, and Rayleigh scattering [11]. The raised cosine interferometric curve and the reciprocal setup lead to output signal ambiguity, which requires a modulation to recover the Sagnac phase that is proportional to the angular rate. The modulation is usually a periodic signal that needs to be synchronously demodulated, requiring fast electronics and trustworthy, linear, and calibrated phase modulators [12].

In the 1980s, it was proposed a nonreciprocal IFOG assembly using a 3 × 3 directional coupler [13]. The ideal 3 × 3 coupler provides an intrinsic phase shift of 2π/3 due to energy conservation [10]. So, instead of the initial phase shift at zero, now we have access to two output signals, one at −2π/3 and the other at 2π/3, that can be used to recover the Sagnac phase shift. However, it is unreasonable to believe that there is a perfectly symmetric 3 × 3 coupler. Moreover, the coupling relation can change with temperature, polarization, and power, resulting in a source of phase drift. This is the biggest difference between the reciprocal and nonreciprocal assemblies: in the traditional reciprocal assembly, the change in coupling relation is ignored because both beams travel through the same optical path. Therefore, the nonreciprocal assembly is not going to achieve better values of bias drift than the reciprocal assembly. On the other hand, it is simpler to implement; it dismisses the phase modulator, requires fewer electronic devices, and works with low-frequency electronic signals. To take advantage of this simplicity, and not aiming to beat the closed-loop IFOG, some works were developed to study demodulation techniques that could reduce the effect of the 3 × 3 phase drift [14,15,16,17,18,19,20].

Following this line, in this work, we used the nonreciprocal IFOG configuration to apply and test the SMO on a Sagnac interferometer [7]. This allows the combination of IFOG and nonlinear control theory, expanding the applications mentioned previously. This SMO has characteristics like a Kalman filter that is well suited for navigation signals [21], unlike purely trigonometric signal recovery, such as the arc-tangent method. The nonreciprocal configuration in this work employed a 3 × 3 fiber coupler, eliminating the need for optical phase modulators, such as those based on piezoelectric or electro-optic devices. This is, to the best of our knowledge, the first time a sliding mode control technique has been employed and evaluated for an interferometric fiber-optic gyroscope.

As a consequence of the study on SMO applied to IFOG, an in-depth analysis of the gain tuning for replacing a sign function with a sigmoid was achieved. This presents, for the first time, a discussion that considers the sampling rate in the gain value definition. Also, we propose an iterative simulation method to generate a fitting function to define the sigmoid gain based on the classical sign function gain, the estimated signal frequency, and the chosen sampling frequency. These results may be useful for the application of SMO to other fields, not only to IFOG.

In Section 2, we present in detail the experimental setup, explaining the optical assembly and the electronic post-processing. Section 3 presents the theory for the SMO applied to the IFOG, including a new analytical method to choose the sampling frequency. Section 4 presents the analysis of the sigmoid gain tuning. In Section 5, we present the experimental results and discussion. Finally, in Section 6 we conclude the discussion with suggestions for the next implementations.

### Sliding Mode Control Principles

Linear control is a well-established technique to automatically control a piece of equipment or devices. It is used in a large range of applications due to its theoretical simplicity. However, applying linear control to a device with a nonlinear response can lead to limited performance since the linear control is designed to operate over a limited domain around a linearized region of the transfer function. This means that, in a real-world application, if the system goes out of this region, it can become unstable or operate poorly since it cannot handle the nonlinear response.

On the other hand, a nonlinear control technique presents the following characteristics: the nonlinear control can operate over a nonlinear response, dismissing linearization and in a wider region; the nonlinear control is also able to work under parameter uncertainties; in general, a nonlinear control can generate a simpler system than a linear counterpart; the nature of some system responses does not allow for achieving a linear approximation, which is not a limitation for the nonlinear control [22]. Based on that, it can be noted that the nonlinear control technique presents several advantages over the linear one for applications on nonlinear systems.

Sliding mode control is a type of nonlinear control technique that works by switching the control law between different structures, which makes this controller very attractive due to its implementation simplicity and high robustness. Moreover, it works with arbitrary initial states and with unknown disturbances that do not need to be previously modeled into the system’s plant [1,22].

**Figure 1:**
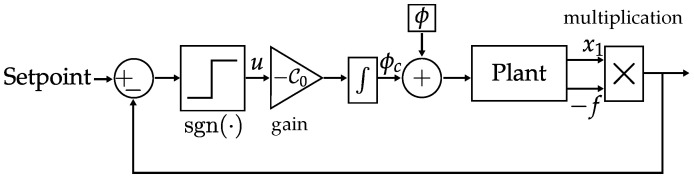
Example of sliding mode control block diagram.

An example of a sliding mode control block diagram is shown in Figure 1, where the plant block represents the model of the system to be controlled. This is a simplified diagram based on the system that will be presented in detail in Section 2. The state variables $x_{1}$ and *f* are multiplied, the product $x_{1}\;\cdot \;f$ is fed back, and it is compared with the setpoint. Thus, the switching mechanism (the sign function) generates a control signal *u* that is amplified by the gain $C_{0}$ and then integrated in time, providing $\phi_{c}$. A disturbance in this diagram is represented by the signal ϕ, which is summed to the integrated control signal and inserted into the plant. In this work, we employed an SMO, which is a state observer based on the sliding mode control principles. In Section 2, the actual SMO used on the IFOG plant (which presents a raised cosine response, i.e., a nonlinear behavior) is presented in detail.

## 2. Experimental Setup

The SMO method proposed in [9] requires two out-of-phase outputs. One way to accomplish this is to mount the IFOG with a 3 × 3 coupler in a passive configuration, without a phase modulator or a multifunction integrated-optic chip (MIOC).

**Figure 2:**
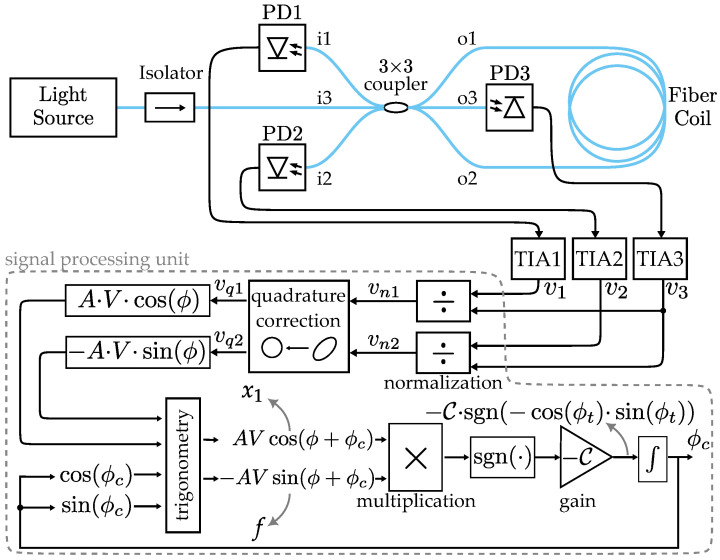
IFOG-SMO experimental setup.

Therefore, the IFOG setup used in this work comprises a light source (superluminescent diode, SLD, mean wavelength λ=1556 nm, Inphenix IPSDD1508, Livermore, CA, USA), an isolator, a 3 × 3 optical fiber coupler, an optical fiber coil, three photodetectors (PD1, PD2, and PD3), and three transimpedance amplifiers (TIA1, TIA2, and TIA3), while the SMO system (implemented in a signal processing unit) contains the following blocks: normalization, quadrature correction, trigonometric calculation, sign function (sgn), gain ($C_{0}$), and integrator, arranged as shown in Figure 2. Although the system can run in real time, as in Felão et al. [9], the TIA signals were logged using a signal acquisition module (NI-9230) with 24-bit resolution for ±30 V full-scale in an National Instruments cRIO-9053 platform, Austin, TX, USA. These voltage signals were sent to a computer, acting as the signal processing unit, used for post-processing.

All optical components employ polarization-maintaining (PM) optical fibers (except photodetectors) to eliminate polarization fading problems and correctly assess the observer’s capability. The coil length and diameter were L=582.8 m and D=140 mm, respectively, employing a bow-tie optical fiber (Fibercore HB1500G-RT, Southampton, UK, cladding diameter d=174 μm), with a quadrupolar winding pattern (8 layers and 164 turns per layer).

The SLD delivers light to the isolator (used to avoid light returning to the source), which delivers light to the 3 × 3 coupler input central fiber (i3). The 3 × 3 coupler divides the light source amplitude into three equal parts, where outputs o1 and o2 are connected to the fiber coil and output o3 is connected to PD3. Thus, the light that enters o1 and o2 travels the coil in both clockwise and counterclockwise directions, recombining at the 3 × 3 coupler. The interferometer output is then delivered to i1, i2, and i3 input ports, where i1 is connected to PD1 and i2 is connected to PD2. Although the light amplitudes are equally divided by the 3 × 3 coupler, the relative phases between the light at i1 and i2 are shifted by 2π/3. In this way, the 3 × 3 coupler provides the two out-of-phase interferometric signals required by the SMO configuration used in this work.

The theoretical transfer functions for a Sagnac interferometer using a 3 × 3 coupler, in terms of the output optical power at PD1 and PD2, are given by

(1) $$\begin{array}{c} P_{1}\left(t\right)=2\;\cdot \;P_{0}/9\;\cdot \;\left\{1+V_{1}\;\cdot \;\cos\left[\phi \left(t\right)-2\pi /3\right]\right\}, \end{array}$$

(2) $$\begin{array}{c} P_{2}\left(t\right)=2\;\cdot \;P_{0}/9\;\cdot \;\left\{1+V_{2}\;\cdot \;\cos\left[\phi \left(t\right)+2\pi /3\right]\right\}, \end{array}$$

where $P_{0}$ is the light source input optical power, $V_{1}$ and $V_{2}$ are the interferometer visibilities (or contrast), and ϕ(t) is the Sagnac interferometer phase given by

(3) $$\phi \left(t\right)=\frac{2\pi \;\cdot \;L\;\cdot \;D}{\lambda \;\cdot \;c}\;\cdot \;\;\Omega \left(t\right),$$

where *L* is the optical fiber coil length, *D* is the coil diameter, λ is the light source mean optical wavelength, and Ω(t) is the rotation rate to be measured. The constant term corresponds to the scale factor

(4) $$K=\frac{2\pi \;\cdot \;L\;\cdot \;D}{\lambda \;\cdot \;c},$$

that converts the rotation rate to the optical phase.

Equations (1) and (2) provide the output optical power that reaches photodetectors PD1 and PD2, which, in turn, convert the optical power into electrical current (through the photodetector responsivity, R), while the transimpedance amplifiers (TIA) convert the current to voltage (through the resistor, *R*). Thus, the interferometric voltage signals are given by

(5) $$\begin{array}{cc} v_{1}\left(t\right) & =R_{1}\;\cdot \;R_{1}\;\cdot \;2\;\cdot \;P_{0}/9\{1+V_{1}\cos\left[\phi \left(t\right)\right]\}\;\;\;\;\;\; \end{array}$$

(6) $$\begin{array}{cc} v_{2}\left(t\right) & =R_{2}\;\cdot \;R_{2}\;\cdot \;2\;\cdot \;P_{0}/9\left\{1+V_{2}\cos\left[\phi \left(t\right)+\theta \right]\right\}, \end{array}$$

where $R_{1}$ and $R_{2}$ are the transimpedance resistors for TIA1 and TIA2, respectively, $R_{1}$ and $R_{2}$ are the responsivity of PD1 and PD2, respectively, and θ is the phase shift between $v_{1}\left(t\right)$ and $v_{2}\left(t\right)$. In addition, the voltage for power normalization is obtained from PD3 and TIA3, given by $v_{3}\left(t\right)=R_{3}\;\cdot \;R_{3}\;\cdot \;P_{0}/3$.

## 3. Sliding Mode Observer for IFOG

At this stage, the voltage signals $v_{1}\left(t\right)$, $v_{2}\left(t\right)$, and $v_{3}\left(t\right)$ are inserted into the signal processing unit, whose role is to acquire and digitize the signals, to implement the SMO, and to provide the rotation rate measurement.

To avoid the influence of eventual light source power fluctuations, signals $v_{1}\left(t\right)$ and $v_{2}\left(t\right)$ are normalized by $v_{3}\left(t\right)$ in the normalization block as follows:

(7) $$\begin{array}{cc} v_{n_{1}}\left(t\right)=\frac{v_{1}\left(t\right)}{v_{3}\left(t\right)} & =A_{1}\;\cdot \;\left\{1+V_{1}\;\cdot \;\cos\left[\phi \left(t\right)\right]\right\},\;\;\;\; \end{array}$$

(8) $$\begin{array}{cc} v_{n_{2}}\left(t\right)=\frac{v_{2}\left(t\right)}{v_{3}\left(t\right)} & =A_{2}\;\cdot \;\left\{1+V_{2}\;\cdot \;\cos\left[\phi \left(t\right)+\theta \right]\right\}. \end{array}$$

where $A_{1}=R_{1}\;\cdot \;R_{1}\;\cdot \;2/3\;\cdot \;R_{3}\;\cdot \;R_{3}$ and $A_{2}=R_{2}\;\cdot \;R_{2}\;\cdot \;2/3\;\cdot \;R_{3}\;\cdot \;R_{3}$. This normalization is computed continuously during the IFOG operation to remove the influence of $P_{0}$ variation.

The normalized signals $v_{n_{1}}\left(t\right)$ and $v_{n_{2}}\left(t\right)$ enter the quadrature correction block that fits an ellipse between these two signals to provide the calibration parameters θ, $A_{1}$, $A_{2}$, and $A_{1}V_{1}/A_{2}V_{2}$. These parameters are then used to convert the two arbitrarily out-of-phase signals, $v_{n_{1}}\left(t\right)$ and $v_{n_{2}}\left(t\right)$, to in-phase and quadrature-phase signals:

(9) $$\begin{array}{cc} v_{q_{1}}\left(t\right) & =v_{n_{1}}\left(t\right)-A_{1},\;\;\;\;\;\;\;\;\;\;\;\;\;\;\;\;\;\;\;\;\;\;\;\;\;\;\;\;\;\;\;\;\;\;\;\;\;\;\;\;\;\;\;\;\;\;\;\;\;\;\;\;\;\;\;\;\;\;\;\; \end{array}$$

(10) $$\begin{array}{cc} v_{q_{2}}\left(t\right) & =\frac{A_{1}V_{1}}{A_{2}V_{2}}\;\cdot \;\frac{\left[v_{n_{2}}\left(t\right)-A_{2}\right]}{\sin(\theta )}-\left[v_{n_{1}}\left(t\right)-A_{1}\right]\;\cdot \;\cot\left(\theta \right). \end{array}$$

Resulting in

(11) $$\begin{array}{cc} v_{q_{1}}\left(t\right) & =A\;\cdot \;V\;\cdot \;\cos[\phi (t)],\; \end{array}$$

(12) $$\begin{array}{cc} v_{q_{2}}\left(t\right) & =-A\;\cdot \;V\;\cdot \;\sin[\phi (t)], \end{array}$$

where $A_{1}\;\cdot \;V_{1}=A\;\cdot \;V$. This quadrature correction must be calculated only once in order to calibrate the system. The quadrature signals $v_{q_{1}}\left(t\right)$ and $v_{q_{2}}\left(t\right)$ are inserted into the trigonometric calculation block of the closed-loop system, which provides the following state variables [9], as shown in Figure 2:

(13) $$\begin{array}{cc} x_{1}\left(t\right) & =A\;\cdot \;V\;\cdot \;\cos[\phi \left(t\right)+\phi_{c}\left(t\right)], \end{array}$$

(14) $$\begin{array}{cc} f(t) & =-A\;\cdot \;V\;\cdot \;\sin[\phi \left(t\right)+\phi_{c}\left(t\right)]. \end{array}$$

The product $x_{1}\left(t\right)\;\cdot \;f\left(t\right)$ is then inserted in the sign function block, which switches between a plus or minus signal according to the product signal. The gain block provides a control gain to obtain the SMO control law, given by

(15) $$u\left(t\right)=-C_{0}\;\cdot \;\mathrm{sgn}\left[x_{1}\left(t\right)\;\cdot \;f\left(t\right)\right]={\dot{\phi }}_{c}\left(t\right),$$

where $C_{0}$ is the control gain and sgn(a)=a/|a| is a sign function. The integrator block provides the control signal through the mathematical integration of u(t), as follows:

(16) $$\phi_{c}\left(t\right)=\int u(t)\;\cdot \;dt.$$

Finally, the control signal $\phi_{c}\left(t\right)$ is fed back to the trigonometric calculation block, thereby closing the loop of the observer. Therefore, the output signal is the control signal itself, which contains the measurement of interest, because $\phi_{c}\left(t\right)=-\phi \left(t\right)+\frac{k\pi }{2}$, where *k* is odd.

### 3.1. Sliding Mode Stability Analysis

The sliding mode observer stability can be proved by Lyapunov’s method, using the state variables $x_{1}$, *f*, (13), and (14). Choosing the sliding surface as $s=x_{1}$, a Lyapunov candidate function might be $V=s^{2}/2$. The analysis is carried out considering an open ball with radius π/2 in every odd equilibrium point multiple of π/2. Then, the Lyapunov candidate function can be confirmed to be positive definite, and its time derivative to be negative definite. Therefore, due to the function periodicity, *V* is shown to be the Lyapunov function for this system, and the equilibrium points are locally asymptotically stable. Refer to Quispe-Valencia et al. [23] for this stability proof using a rigorous mathematical formalism.

**Figure 3:**
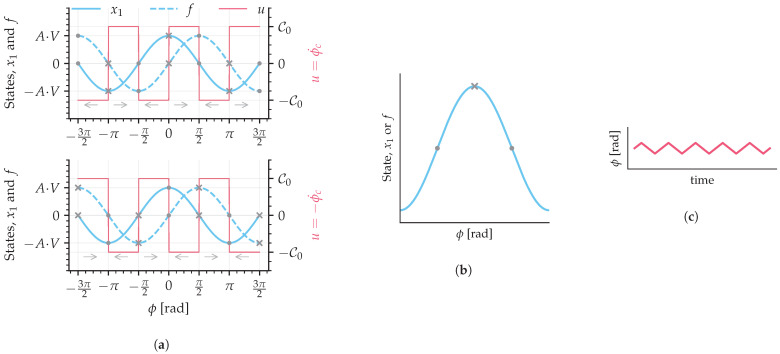
Sliding mode representation of switching mechanism. (**a**) State variables with switching control signal *u*. (**b**) Example of a single state and its stable equilibrium points. (**c**) Output optical phase shift with a chattering effect. Dots are stable and crosses are unstable equilibrium points; arrows indicate phase direction.

The controller dynamics can be explained by visually examining the state variables and the switching control signal. In Figure 3a, both state variables are shown with their respective equilibrium points; gray dots are stable and gray crosses are unstable. The switching of signal *u*, (15), which is integrated in time, carries the state variables to zero by adding or subtracting a phase (gray arrows indicate phase direction). An interesting fact is that even if the switching signal *u* is inverted, the system is still stable, because a similar analysis is possible with the state *f* instead of $x_{1}$ (Figure 3a bottom plot). Let us analyze the state $x_{1}$ around the equilibrium point π/2 (Figure 3a top plot): whenever the state is bigger than zero, the switching signal is positive and the phase increases, taking the state to a lower value, and analogously for the negative side. Its behavior resembles the operation of a sigma-delta analog-to-digital converter.

The control gain, $C_{0}$, affects the velocity of the control phase shift. By inspecting one of the stable equilibrium points of a state variable (Figure 3b) and its resultant control phase shift (Figure 3c), the chattering phenomenon can be visualized. Since the frequency of the switching signal is finite, the resultant phase might overshoot the stable point, generating the chattering effect.

### 3.2. Chattering Phenomenon

In the practical implementation of this observer system, the sign function may produce the chattering phenomenon, which consists of oscillations with finite frequency and amplitude [1]. To reduce chattering, the sign function can be replaced by a continuous function, such as the sigmoid

(17) $$\mathrm{sgm}\left(a\right)=\frac{a}{|a|+\varepsilon }\;\;,$$

where ε is the positive sigmoid factor. In this work, ε=0.1 and sigmoid gain C=542 were chosen using the sigmoid gain tuning method explained in Section 4.

Recently, a new branch on the research of nonlinear control for interferometry has been proposed by Coradini et al. [24], where the authors applied a Takagi–Sugeno fuzzy nonlinear observer system to a Michelson interferometer, showing the ability to avoid the chattering problem caused by SMO. Although this is not within the scope of the present work, it shows that the nonlinear observers applied to interferometers are still a relevant research topic.

### 3.3. Calibration

**Figure 4:**
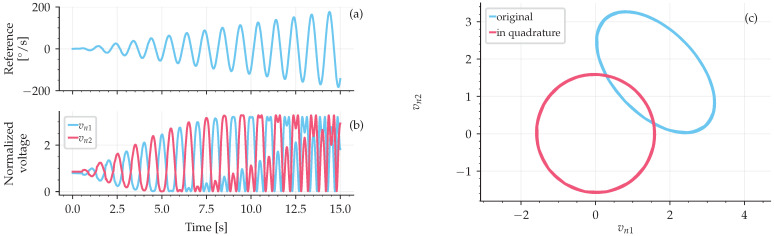
Example of data used to determine the calibration parameters. (**a**) Rotation rate imposed on the IFOG. (**b**) Normalized output signals $v_{n_{1}}\left(t\right)$ and $v_{n_{2}}\left(t\right)$. (**c**) Lissajous figures before and after the parameter calibration.

The first step in using the observer developed in this work was to obtain the calibration parameters. This can be accomplished by submitting the IFOG to a rotation rate, as shown in Figure 4a and acquiring the normalized output signals $v_{n_{1}}\left(t\right)$ and $v_{n_{2}}\left(t\right)$, as shown in Figure 4b. These two output signals of Figure 4b are then used to plot a Lissajous figure that is fitted by an ellipse using the least squares method to find the calibration parameters.

The ellipse fit from the Lissajous figure, Figure 4c, is accomplished following the derivation from [25], which is described in more detail by [26]. Taking a digitized array of signals $v_{n_{1}}\left(t\right)$ and $v_{n_{2}}\left(t\right)$ with *k* samples in the form of (7) and (8), one can perform an algebraic manipulation to obtain

(18) $$A\;\cdot \;v_{n_{1}}^{2}+B\;\cdot \;v_{n_{2}}^{2}+C\;\cdot \;v_{n_{1}}\;\cdot \;v_{n_{2}}+D\;\cdot \;v_{n_{1}}+E\;\cdot \;v_{n_{2}}=1.$$

Constructing the matrices

(19) $$X_{k\times 5}=\left[\begin{array}{ccccc} v_{n_{1}}^{2} & v_{n_{2}}^{2} & v_{n_{1}}\;\cdot \;v_{n_{2}} & v_{n_{1}} & v_{n_{2}} \end{array}\right],$$

(20) $$Q_{5\times 1}={\left[\begin{array}{ccccc} A & B & C & D & E \end{array}\right]}^{T},$$

and

(21) $$I_{k\times 1}={\left[\begin{array}{cccc} 1 & 1 & \ldots & 1 \end{array}\right]}^{T},$$

one can find the values for *A*, *B*, *C*, *D*, and *E* by least squares method:

(22) $$Q={\left(X^{T}\;\cdot \;X\right)}^{-1}\;\cdot \;X^{T}\;\cdot \;I.$$

Then, the fitted parameters are used to transform (7) and (8) to (11) and (12) by calculating

(23) $$\frac{A_{1}V_{1}}{A_{2}V_{2}}=\sqrt{\frac{B}{A}},\;\;\theta =\sin^{-1}\left(\frac{C}{2A}\;\cdot \;\frac{A_{2}V_{2}}{A_{1}V_{1}}\right),\;\;A_{1}=\frac{2B\;\cdot \;D-E\;\cdot \;C}{C^{2}-4A\;\cdot \;B},\;\;\mathrm{and}\;\;A_{2}=\frac{2A\;\cdot \;E-D\;\cdot \;C}{C^{2}-4A\;\cdot \;B}.$$

The number of samples *k* depends on the input signal shown in Figure 4a. Ideally, a modulating signal during the calibration is high enough to complete a full ellipse. In this case, the modulation signal for the calibration was the Sagnac phase by applying an angular rate to the IFOG-SMO.

The fitted parameters in this work were the following: θ=119.74°, $A_{1}=1.84\;V$, $A_{1}=1.88\;V$, $A_{1}V_{1}/A_{2}V_{2}=0.982$. By using these calibration parameters and (9) and (10), the original Lissajous ellipse is converted to a circle (in quadrature) centered at (0,0), as shown in Figure 4c. The code used here to simulate the SMO signal processing unit, including this ellipse fitting, is available as Code C1 from Supplementary Materials as a standalone Julia Programming Language package, version 1.7.

Therefore, from this point on, the IFOG is in quadrature and calibrated to measure rotation rate. The coupling ratio of a standard single-mode (SM) 3 × 3 coupler can vary by up to 3% over the temperature range of −25 °C to 85 °C [18], which also leads to a change in the phase shift. In [14], a passive IFOG with an SM 3 × 3 fiber coupler and the need for recalibration at each operating temperature was reported. Alternatively, in the present work, a PM 3 × 3 fiber coupler was used, and to evaluate the influence of temperature on the IFOG calibration, the static calibration was repeated at 10 °C, 15 °C, 20 °C, and 25 °C, showing a phase variation of less than 0.2%, which is low when compared with references [13,14]. This justifies the use of the PM 3 × 3 fiber coupler in this work, instead of the SM 3 × 3 fiber coupler used in [14,17,18,19].

### 3.4. Minimum Sample Rate

The minimum sample rate influences the final cost and implementation complexity of the acquisition system and the signal processing design. It is not sufficient to satisfy the Nyquist criteria when selecting the sample rate for IFOG-SMO. The interferometric signal represents the optical phase difference between the light beams; therefore, the sample rate requirement considers both the frequency and amplitude of the signal to correctly sample all interferometric fringes.

In order to estimate the sampling frequency necessary to correctly acquire the signal, a sinusoidal-shaped angular rate was chosen (without loss of generality, since any arbitrary periodic signal can be decomposed by a Fourier series), as follows:

(24) $$\Omega \left(t\right)=\Omega_{0}\;\cdot \;\sin\left(2\pi \;\cdot \;f\;\cdot \;t\right).$$

Then, the signal of interest becomes

(25) $$\phi \left(t\right)=K\;\cdot \;\Omega_{0}\;\cdot \;\sin\left(2\pi \;\cdot \;f\;\cdot \;t\right),$$

where $\Omega_{0}$ is the angular rate amplitude, *f* is the angular rate frequency, and *t* is time. For this signal of interest, the number of fringes generated is $N=K\;\cdot \;\Omega_{0}/2\pi$. Then, considering the angular frequency of this periodic angular rate signal as ω=2πf, and the desired number of points for a single fringe as *p*, the sample rate can be calculated by

(26) $$\begin{array}{c} f_{s}=N\;\cdot \;\omega \;\cdot \;p=K\;\cdot \;\Omega_{0}\;\cdot \;f\;\cdot \;p. \end{array}$$

We found empirically that 50≤p≤100 is suitable for this application. For example, the minimum sample rate can be calculated using the maximum expected experimental values applied to the IFOG of K=1.1 s, $\Omega_{0}=1000$°/s, f=1 Hz, and p=50. This results in a minimum sample rate of approximately 960 Hz, and then we used 1 kHz.

## 4. Sigmoid Gain Tuning

According to Felão et al. [9], the control gain required for the SMO to meet the reachability criterion should be greater than the rate of interferometric phase variation:

(27) $$C_{0}>\left|\dot{\phi }\right|.$$

Thus, considering a signal of interest as (25), the control gain value required to guarantee the reachability criterion is chosen according to

(28) $$C_{0}>K\;\cdot \;\Omega_{0}\;\cdot \;2\pi f.$$

This gain, $C_{0}$, only holds for a system employing the sign function; it is not theoretically specified for the case where the sign function is replaced by the sigmoid function (17). The sigmoid gain is now referred to as C because it is different from $C_{0}$ of (28).

Although various continuous functions are widely used in the literature as substitutes for the sign function, the drawbacks of this substitution, such as increased gain, higher sampling rates, or both, have rarely been discussed. The gain value and sample rate shall be chosen with compromises, that is, a gain high enough to obey the reachability criterion with an acceptable level of chattering for a given application and a minimum possible sample rate to reduce electronics costs and complexity.

Some studies have explored the use of different sigmoid functions as substitutes for the sign function in SMO. For instance, in [27], the authors compared different sigmoid shapes for SMO applied to a synchronous motor. They considered the saturation, exponential sigmoid, and hyperbolic functions. The hyperbolic function performs the worst in terms of the error noise level, whereas the other two functions are equivalent. In [28], the gain for the saturation function is given as the sign gain times the inclination of the function. In [29], the gain for the exponential function is given as the sign gain times the sigmoid function of the error. In [30], besides comparing different sigmoid function shapes, gain adaptation was proposed to reduce the dynamic error and oscillation. In [31], proportional-integral-derivative control principles were applied to adapt the sign function gain instead of replacing it with a sigmoid function.

Therefore, in this work, we propose to accomplish a computational simulation to evaluate the optimal gain required while obeying the reachability criterion for a sigmoid function of the type (17); this approximation of the sigmoid function was chosen due to its computational efficiency [30]. Furthermore, this analysis extends to the setting of the ε value, that is, evaluating the cases where the sigmoid shape changes from the shape of the sign function (ε=0) to a smoother shape (ε>0). Additionally, multiple sample rates were considered for each value of ε.

**Figure 5:**
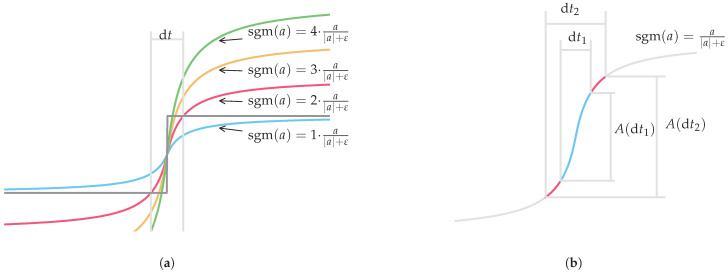
(**a**) Example of sigmoid gain difference compared with sign function. (**b**) Example of sigmoid gain difference due to sample rate.

A visual representation of different gains for the same sigmoid function is presented in Figure 5a, which shows that for a fixed sample period d*t*, the maximum amplitude reached by the sigmoid function differs. The effect of using different sample rates for the same sigmoid function with the same gain is presented in Figure 5b, which shows a similar effect in the final amplitude of the sigmoid function. A faster sample rate results in a smaller sample period, which in turn results in a smaller sigmoid output amplitude. However, the switching speed is faster, which might compensate for the smaller amplitude. This is considered in an extensive simulation of parameters that resulted in a transfer function that can be used to tune the sigmoid gain depending on the sample rate ($f_{s}$), sigmoid factor (ε), and the sign function gain ($C_{0}$), which is calculated based on the expected input signal frequency (*f*) and amplitude ($K\;\cdot \;\Omega_{0}$).

The simulation was performed by employing a sinusoidal rotation rate (24) and by inserting (4) in (3) to obtain $v_{1}\left(t\right)$ and $v_{2}\left(t\right)$ with (5) and (6). Note that Ω(t) represents the upper limit rotation rate that can be tracked by the observer, i.e., it should reflect the more stringent rotation rate to be imposed on the IFOG. Then, $v_{1}\left(t\right)$ and $v_{2}\left(t\right)$ can be inserted in the observer system, which, in turn, provides the output signal $\phi_{c}\left(t\right)$, used to calculate the measured rotation rate, as $\Omega_{m}\left(t\right)=-\phi_{c}\left(t\right)/K+k\pi /2$.

We used the specific input values for amplitude of $K\;\cdot \;\Omega_{0}=1\;\mathrm{rad}$ and normalized frequencies ($f/f_{s}$) from $5\times 10^{-4}$ to $10^{-2}$ for the simulations.

**Figure 6:**
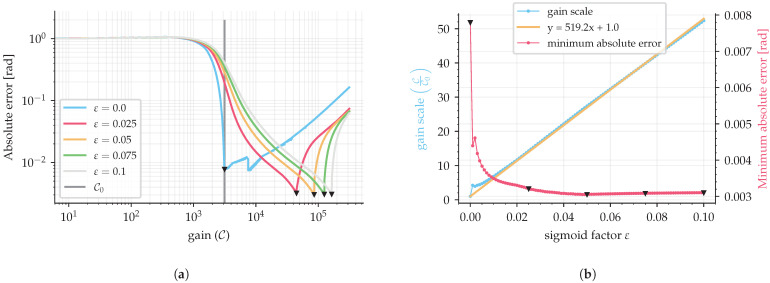
SMO simulation with a sine wave input signal of 500 Hz and a sampling period of 2 μs ($f/f_{s}=0.001$). (**a**) is the error due to different gains, $C_{0}$, for different sigmoid factors and (**b**) is the information of optimal gains from (**a**). Triangles indicate the point of minimum for each absolute error curve.

**Figure 7:**
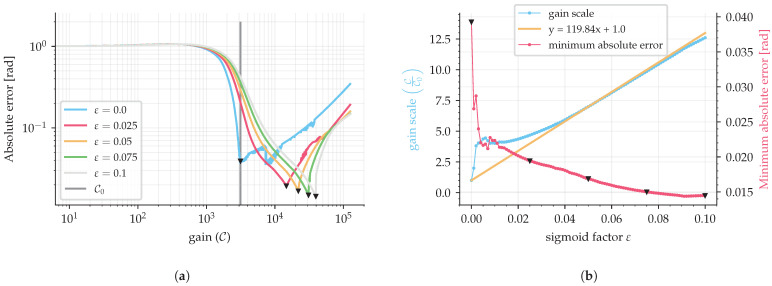
SMO simulation with a sine wave input signal of 500 Hz and a sampling period of 10 μs ($f/f_{s}=0.005$). (**a**) is the error due to different gains, $C_{0}$, for different sigmoid factors and (**b**) is the information of optimal gains from (**a**). Triangles indicate the point of minimum for each absolute error curve.

To evaluate the magnitude of the chattering phenomenon and, simultaneously, to evaluate the error caused by the lack of control tracking (causing the system to be unstable), one can calculate the absolute phase error between the input rotation rate and the measured rotation rate. The absolute phase error was then evaluated as

$$\Gamma =K\;\cdot \;(\Omega \left(t\right)-\Omega_{m}\left(t\right))$$

as a function of the control gain value, for a given sigmoid factor ε, thereby providing a respective curve, as shown in Figure 6a and Figure 7a. This curve presents a minimum absolute error point, where one can select the optimal control gain (point of minimum absolute error) for each sigmoid factor ε. The final transfer function of sigmoid gain tuning is then obtained by analyzing the optimal gains for different sigmoid factors for multiple sample rates.

## 5. Results and Discussion

### 5.1. Simulation Results

Based on the method described in Section 4, a simulation was performed to obtain a series of curves for the absolute error Γ as a function of the control gain C. It was simulated for sigmoid factors in the interval between 0 and 0.1, with steps of 0.001, and some examples of these curves are shown in Figure 6a. A sine wave with f=500 Hz and $K\;\cdot \;\Omega_{0}=1\;\mathrm{rad}$ was used as the input signal, and the simulation was performed with a sampling period of 2 μs.

The minimum value for each curve represents the optimal gain value, where the system is stable and without extra chattering. From these data, another graph was plotted to provide a view of the optimal gain value and the minimum absolute error, both as a function of the sigmoid factor, as shown in Figure 6b. A linear increase tendency of the optimal gain is noticeable as a function of the sigmoid factor. While the absolute error decays exponentially, approximately 50% for a sigmoid factor from 0 to 0.02. This indicates the benefit of using the sigmoid function, which, at the expense of increasing the gain linearly, causes the absolute error to decay exponentially. In addition, the absolute error remains at low values for sigmoid factors above ε>0.05.

Then, repeating the same procedure but using a sampling period of 10 μs resulted in Figure 7a,b, which presented the same behavior as the 2 μs sampling period.

**Figure 8:**
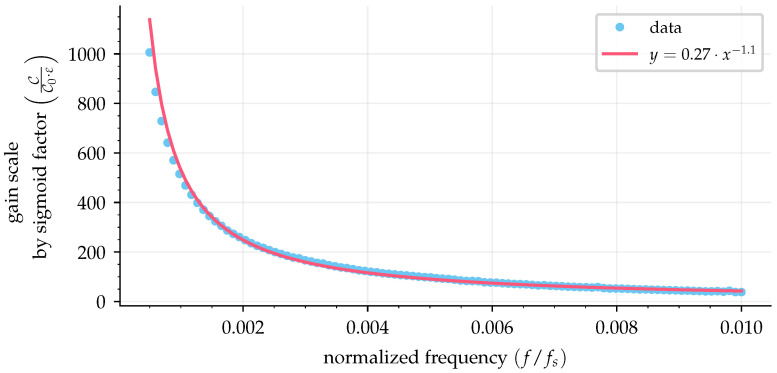
Gain scale factor by normalized frequency.

It is necessary to analyze the effect of the sampling period on the optimal gain. Note that Figure 6a and Figure 7a have different optimal gain values for the same sigmoid factor because different sample periods were used in the simulations. A faster sampling rate requires a higher gain because the sigmoid function is not instantaneous as the sign function, which results in a fraction of the gain value for the feedback loop, as shown in Figure 5b. To demonstrate this, 100 simulations with normalized frequencies ranging from $5\;\times \;10^{-4}$ to $10^{-2}$ (500 Hz input signal with sampling periods from 10μs to 0.5μs) were used to simulate curves of absolute error as a function of gain (as in Figure 6b and Figure 7b). Fitting the best line by least squares on these curves provided a slope value with information of gain scale ($C/C_{0}$) compared with the sign function by the value of the sigmoid factor (ε); then these slopes were used to make Figure 8.

The resulting curve in Figure 8 shows the angular coefficient of the gain scale by the sigmoid value ($C/C_{0}/\varepsilon$) by the normalized frequency ($f/f_{s}$). The best fit for the curve indicated a power law form. This curve can be used to correct the gain value when a sigmoid is used in the sliding mode observer and is presented as

(29) $$\frac{C}{C_{0}\;\cdot \;\varepsilon }=m\;\cdot \;{\left(\frac{f}{f_{s}}\right)}^{-n},$$

where m=0.27 and n=1.1. For example, when using a sign function, the required gain to reach stability according to (28) is $C_{0}$, and for a normalized frequency of $10^{-3}$, one can find the value for the gain scale by a sigmoid factor of ∼500 in Figure 8. Considering a sigmoid factor of ε=0.1, the new gain must be $C\approx 50\;\cdot \;C_{0}$.

**Figure 9:**
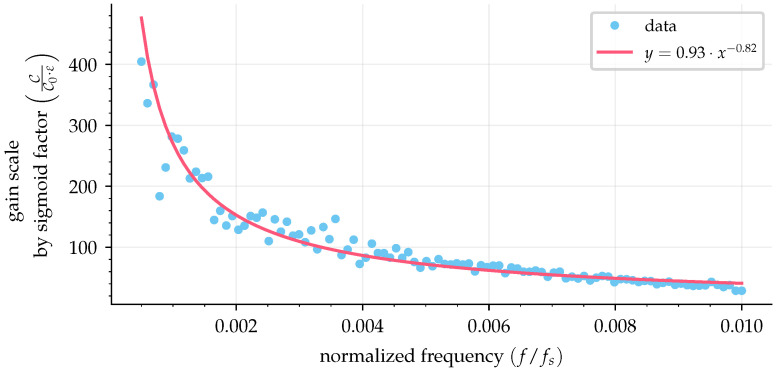
Gain scale factor by normalized frequency considering random noise.

It is important to note that the parameters fitted from the curve in Figure 8 and presented by (29) are dependent on the numerical integrator used in the SMO. In this case, the same integrator was used in the simulations and the experiments, that is, the Bogacki–Shampine method (BS3). In addition, to match the experimental data, the simulation was repeated with random noise with an amplitude of −50 dB added to the synthesized input signal, as shown in Figure 9, with m=0.93 and n=−0.82.

These results are very useful for practical applications because the implementation of the sliding mode observer does not need to be guessed. A simple estimate of the input signal amplitude and frequency allows us to determine the required gain and sampling frequency.

### 5.2. Experimental Results

To prove the concept and show the measurement of the rotation rate properly, two experiments were conducted: one to show the measurement of high rotation rates (in this case, up to 1000°/s) and the second to show small rotation rate measurements. These experiments were conducted on a rotary table (Acutronic 1-Axis BD125, precision of 10μdeg/s) inside a controlled thermal chamber at 20 °C.

**Figure 10:**
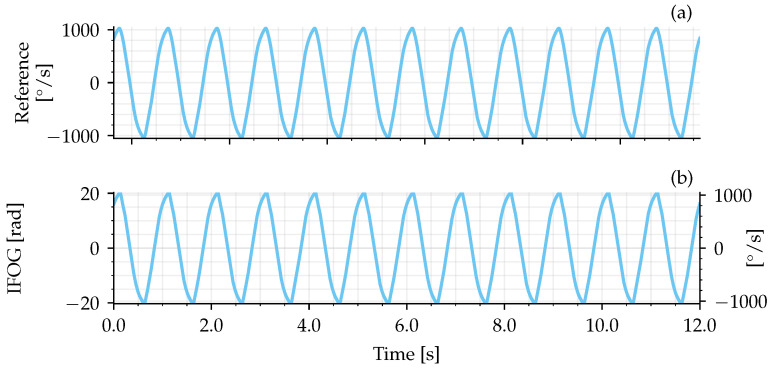
Rotation rate example signal recovered by IFOG-SMO. (**a**) Rotation rate imposed by the rotary table, measured with the table encoder. (**b**) Rotation rate measured with the IFOG-SMO.

The first experiment was performed by submitting the IFOG-SMO to a rotation rate varying in sinusoidal semi-cycles with 1000°/s amplitude and 1 Hz, as shown in Figure 10a. The IFOG-SMO measured signal, ϕ(t), is presented in Figure 10b. Comparing these two signals, it can be noted that the system was able to properly recover the rotation rate waveform and to measure up to 1000°/s.

**Figure 11:**
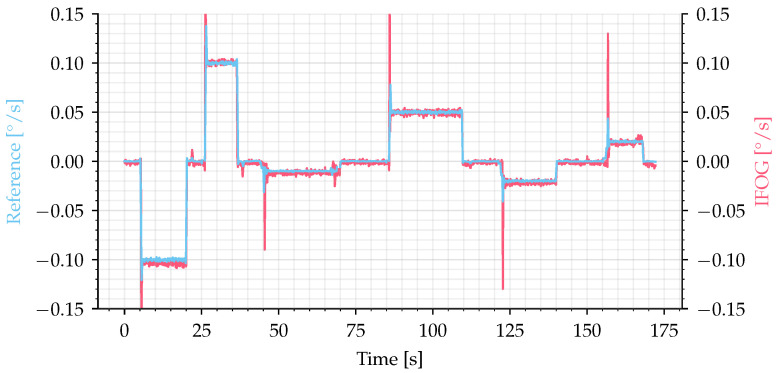
Small values of rotation rate recovered by IFOG-SMO, with −0.1, +0.1, −0.01, +0.05, −0.02, +0.02°/s. Source: adapted from [32].

The second experiment was conducted by applying rotation rate in steps of −0.1, +0.1, −0.01, +0.05, −0.02, and +0.02°/s, as shown in Figure 11 by the blue line. The output signal (filtered by a 5 Hz second-order filter) is shown by the red line, where it can be noted the correct measurement of the small angular velocities applied to the IFOG-SMO.

**Figure 12:**
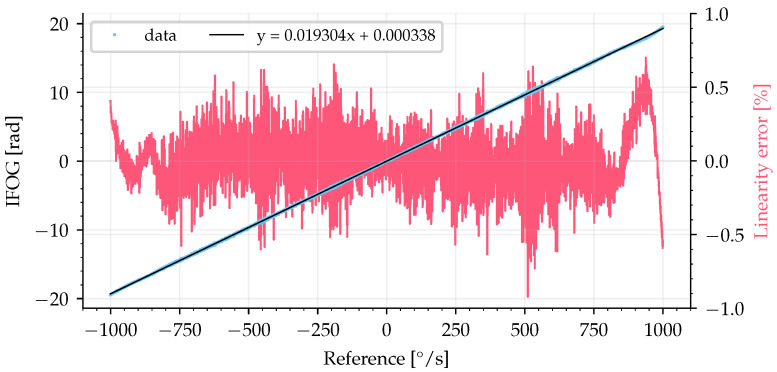
Linearity curve and linearity error calculated up to 1000°/s with a signal as shown in Figure 10. Blue dots are the experimental averaged values, black line is a linear fit, and red line is the linearity error.

The data for measuring the linearity error and the scale factor were acquired from 184 full cycles (as shown in Figure 10) to calculate the average around integer values of rotation rate (with a step of 1°/s). The linearity curve, shown in Figure 12, was plotted using the experimental averaged values (blue dots) with a linear fit using the least squares method (black line), and the linearity error (red line) was calculated.

The linearity error, ξ, was calculated as the absolute difference between the averaged experimental data and the linear fit divided by the difference between upper (+1000°/s) and lower (−1000°/s) limits (full range of 2000°/s), according to [33]:

(30) $$\xi =\frac{x-x_{\mathrm{fit}}}{x_{\mathrm{upper}}-x_{\mathrm{lower}}}=\frac{x-x_{\mathrm{fit}}}{2000}.$$

The linearity error had a maximum error of approximately 0.5%, equivalent to approximately 0.2 rad, in the interval from −1000°/s to +1000°/s. The code used for linearity error calculation is available as Code C2 from Supplementary Materials.

**Figure 13:**
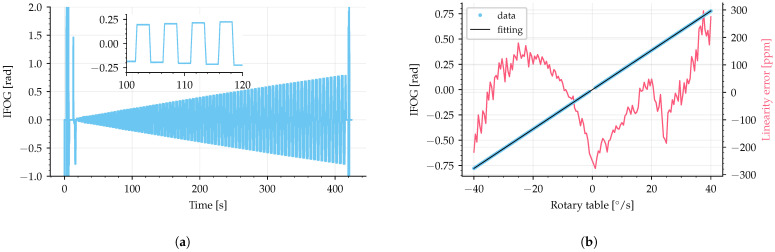
Linearity error up to 40°/s. (**a**) shows the signal measured by the IFOG-SMO and (**b**) shows the linearity curve and the linearity error (blue dots are the experimental averaged values, black line is a linear fit, and red line is the linearity error).

The method used to acquire the linearity error in Figure 12 was accomplished by using a dynamic input signal. This means that it requires a short characterization time to obtain a large sample of input versus output data points. That is, in a single sinusoidal signal, we obtain the full range of the sensor instead of quantizing the signal in steps, which would require more time to sweep the entire full range. The perfect synchronization between the input and output data is difficult to achieve in practice. Any sample delay between the input and output data increases the linearity error. Therefore, the preferred method in the literature to characterize linearity error is to set a fixed angular rate as input for a large amount of time (to guarantee a continuous movement) and average the output signal to obtain a single value as the output signal for each input fixed-step. Thus, the linearity error was calculated using this second method for angular rates up to 40°/s (full range of 80°/s) without temperature control, as shown in Figure 13, which resulted in a maximum linearity error of 300 ppm, approximately 0.5 mrad.

The scale factor measured from the linear fit is 1.1 s (by converting the rotation rate on the x-axis of Figure 12 from °/s to rad/s), which agrees with the calculated one using (4), equal to 1.1, regarding the optical fiber length L=582.8 m, coil diameter D=140 mm, λ=1556 nm, and spare optical fiber length of 6.4 m due to components.

**Figure 14:**
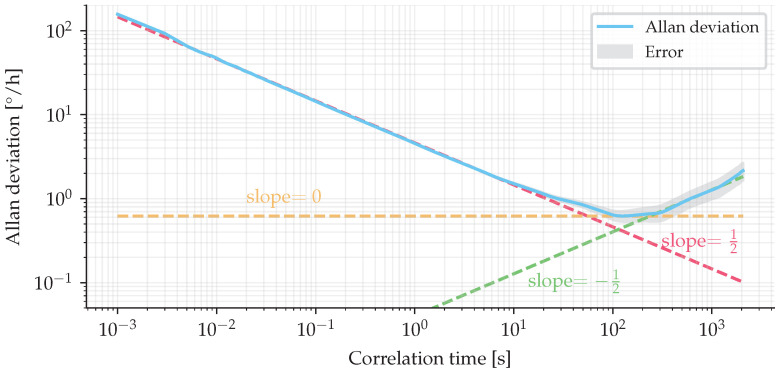
Allan deviation.

Finally, the Allan deviation was measured by inserting the IFOG-SMO in a chamber with the temperature controlled at 20°C, and the result is presented in Figure 14. From these data, the following results were obtained: bias drift of 0.93°/h, angle random walk (ARW) of $76\times 10^{-3}{}^{\circ}/\sqrt{h}$, and rate random walk (RRW) of 4.23°$/h/\sqrt{h}$.

**Figure 15:**
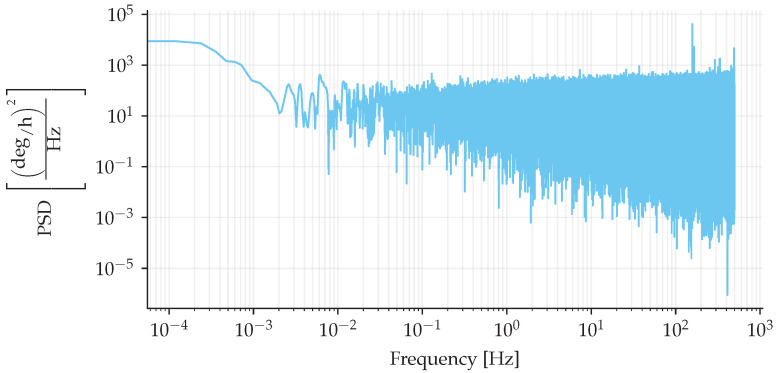
Power spectrum density of IFOG-SMO at rest in a temperature-controlled chamber at 20 °C.

**Figure 16:**
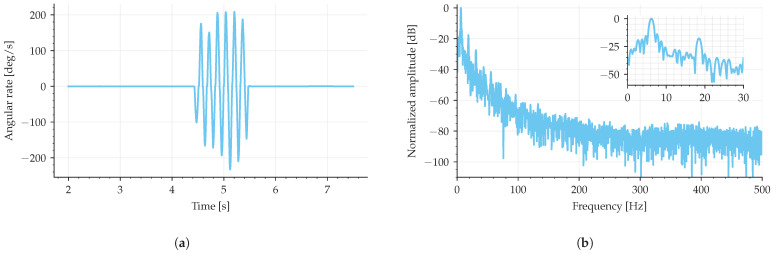
Signal-to-noise ratio. (**a**) shows an angular rate measured by the IFOG-SMO and (**b**) shows its normalized amplitude spectrum.

The same data that generated Figure 14 was used to calculate the power spectrum density (PSD), shown in Figure 15. The PSD shows a distinct disturbance signal around 156 Hz; other than that, no distinct trend was noticed. Also, an arbitrary motion was imposed on the IFOG-SMO by manually rotating it, shown in Figure 16a, and its normalized amplitude spectrum presents a signal-to-noise ratio of 20 dB, shown in Figure 16b.

It is worth mentioning that the IFOG-SMO full scale would be theoretically limited by the coherence length ($L_{c}=c/\Delta \nu$) of the light source used, as λ/Δλ≈44.56πrad≈ 7250°/s. In this experiment, it was limited by the full input range of the rotary table, which was 1000°/s. The dynamic range is calculated using the maximum and minimum values the IFOG-SMO can measure. The bias drift of 0.93°/h can be used as a minimum value for situations of long integration time, and the maximum rotation rate measured was 1000°/s, which is equivalent to phase shifts of 5.22μrad and 6.11π rad, respectively. This results in a dynamic range of 131.3 dB. On the other hand, considering the maximum estimated rotation rate of 7250°/s (which corresponds to 44.56π rad) would provide a dynamic range of 148.5 dB.

Regarding an open-loop IFOG with the same coil, the full scale would be 464°/s, or 2.86π rad, with a dynamic range of 157.1 dB [34], whereas a closed-loop IFOG using the method presented by Bacurau et al. [35] would provide a full scale of 162°/s, or π rad, with a dynamic range of 162.5 dB. The IFOG-SMO dynamic range could be expanded by improving the bias drift value. The comparison of IFOG’s full scale shall be made using the maximum interferometric phase that can be measured. Otherwise, by reducing the coil length or diameter in half, one could argue that the IFOG doubled its full-scale capability. However, the phase in radians is the same as before because of the method employed. Therefore, the results show the expansion of the full-scale input by employing the SMO technique in the IFOG.

The long-term stability is limited by the nonreciprocal construction of the IFOG-SMO, as mentioned in Section 1. So, the 3 × 3 coupler’s phase drift, optical power fluctuation, temperature variation and gradient (Mohr’s and Shupe’s effect), and magnetic field could degrade the long-term stability. This setup was not enclosed in a μ-metal case to protect from magnetic field disturbances. The Kerr effect inside the coil due to power fluctuation causes drift and noise since the normalization from (7) and (8) is for demodulation purposes. As a practical example, for this IFOG-SMO assembly to measure an angular rate of 1°/h, it has to be able to resolve an interferometric phase shift of 5.3 μrad. Therefore, to achieve a bias drift of 0.93°/h without active modulation is a confirmation that the SMO can work with IFOGs and that the gain tuning, the sigmoid factor, and the sampling rate were suitably chosen.

The IFOG-SMO developed in this work presented a better performance in comparison to 3 × 3 passive IFOG reported in [14] (bias drift of 4.7°/h, full scale of 200°/s and [15] (bias drift of 144°/h, ARW of 20°/h). Better performance can be noted in comparison with the passive resonant fiber-optic gyroscope (RFOG) presented in [36] (bias drift of 20°/h, ARW of 0.16°/h), while it was surpassed by the passive RFOG of [37] (bias drift of 0.03°/h, ARW of $0.2\;\cdot \;10^{-3}{}^{\circ}/\sqrt{h}$).

## 6. Conclusions

In conclusion, the SMO applied to the IFOG with a 3 × 3 coupler provides simple and low-cost construction since it does not require an optical phase modulator, whether it is a wideband (lithium niobate modulator, as an MIOC) or a narrowband modulator (piezoelectric cylinder). These modulators are relevant components for the performance of a traditional IFOG configuration and usually require more electronic parts, which complicates the design and needs further logic to control the modulation depth. In addition, the IFOG-SMO does not require fast electronics: for instance, we used 1 kS/s for a coil length of 582.8m, while for a closed-loop configuration in [35], a minimum sample rate of 150 kS/s was required for a coil length of 1400m. Thus, a cost saving in the electronics components can be significant for series production [38]. The results show that the IFOG with SMO was able to correctly measure the rotation rate and show the full-scale expansion capability. Usually, short length (*L* below about 1000 m) low scale factor IFOG, relying on classical open-loop or closed-loop demodulation, requires fast electronics and fast analog to digital converters to deal with the short period of transit time. Nevertheless, using the SMO on these short-length IFOGs permits coil size and volume reduction (hence decreasing optical fiber cost, winding time, and complexity) without scaling up the electronics velocity. Conversely, the same SMO can be applied for long-length (*L* above about 1000 m) high-scale factor IFOG to increase the full scale while keeping the large scale factor.

Moreover, we provided a qualitative analysis of the SMO behavior when the sign function is substituted by a sigmoid function. This provides valuable insights into the sigmoid gain dependence not only on the function shape but also proposes an analysis of the sample rate. The obtained power law function allows a sigmoid gain tuning based on expected input signals instead of using a guess process with arbitrarily high gains and fast sample rates. We presented an experimental method to choose the sample rate based on the expected maximum input signal of the IFOG, maximum amplitude, and frequency desired. These parameters are important for the selection of an IFOG off-the-shelf. The gain tuning and sample rate setting methods, obtained in the present work, may be useful for the application of SMO even in other fields, not only to IFOG.

Having proved the concept of SMO working with an IFOG, the next suggested step is to assemble the same setup using only single-mode optical components, including the 3 × 3 coupler, and use an automatic calibration to fit the Lissajous whenever the signal is bigger than a set threshold, as in [39]. This would consider the 3 × 3 phase drift due to environmental changes and calibrate it to quadrature again. One step further is to actively modulate a phase signal big enough to guarantee a constant quadrature recalibration [40]. With the advantage of not requiring a fast or precise modulation, any modulation signal above the signal of interest cut-off frequency may work, even with low-cost and homemade phase modulators [41]. Theoretically, this could remove the nonreciprocity of the 3 × 3 coupler.

## Data Availability

The raw data supporting the conclusions of this article will be made available by the authors on request.

## Supplementary Materials

The following supporting information can be downloaded at: https://doi.org/10.5281/zenodo.12575545, accessed on 23 May 2025, Code C1: Interferometers.jl; https://doi.org/10.5281/zenodo.12575626, accessed on 23 May 2025, Code C2: Linearity.jl.
